# Learning from feedback: Evaluation of dynamic decision-making in virtual reality under various repetitive training frameworks

**DOI:** 10.3389/fpsyg.2022.872061

**Published:** 2022-11-14

**Authors:** Akash K. Rao, Sushil Chandra, Varun Dutt

**Affiliations:** ^1^Applied Cognitive Science Laboratory, School of Computing and Electrical Engineering, Indian Institute of Technology Mandi, Kamand, Himachal Pradesh, India; ^2^Department of Biomedical Engineering, Institute of Nuclear Medicine and Allied Sciences, Defence Research and Development Organization, New Delhi, India

**Keywords:** decision-making, virtual reality, heterogenous training, difficult training, cognitive workload, instance-based learning

## Abstract

Dynamic decision-making involves a series of interconnected interdependent confluence of decisions to be made. Experiential training is preferred over traditional methods to train individuals in dynamic decision-making. Imparting experiential training in physical settings can be very expensive and unreliable. In virtual reality (VR), synthetic environments play a significant role in providing flexible and cost-effective training environments to enhance dynamic decision-making. However, it is still unclear how VR can be used to impart dynamic decision-making training to increase cognitive performance in complex situations. Besides, different repetitive training methods like desirable difficulty framework and heterogeneity of practice have been evaluated on generic cognitive and motor tasks. However, an evaluation of how these repetitive training methods facilitate dynamic decision-making in an individual in a virtual complex environment setting is lacking in the literature. The objective of this study is to evaluate the effect of different repetitive training methods in immersive VR on dynamic decision-making in a complex search-and-shoot environment. In a lab-based experiment, 66 healthy subjects are divided equally and randomly into three between-subject training conditions: heterogenous, difficult, and sham. On Day 1, all the participants, regardless of the condition, executed an environment of a baseline difficulty level. From Days 2 to 7, the participants alternatively executed the novice difficulty and expert difficulty versions of the environment in the heterogenous condition. In difficult conditions, the participants executed the expert difficulty version of the environment from Days 2 to 7. In the sham condition, the participants executed an unrelated VR environment from Days 2 to 7. On Day 8, the participants executed the baseline difficulty version of the environment again in all the conditions. Various performance and workload-based measures were acquired. Results revealed that the participants in the heterogenous and difficult conditions performed significantly better on Day 8 compared with Day 1. The results inferred that a combination of immersive VR environment with repetitive heterogenous training maximized performance and decreased cognitive workload at transfer. We expect to use these conclusions to create effective training environments in VR for imparting training to military personnel in dynamic decision-making scenarios.

## Introduction

As defined by Gonzalez et al. (2005), dynamic decision-making tasks encapsulate a sequence of interdependent decisions made in an environment. The environment then changes as a function of the series of decisions made, independent of the series, or both ways (Edwards, 1962). According to Jenkins et al. (2011), accurate and coherent decision-making is imperative to ensure safety, accuracy, and efficiency in complex and dynamic systems. Decision-making activities are usually linked to an individual’s expertise in the said domain and their repertoire of rule-based heuristics and ‘if-then’ procedures (Jenkins et al., 2011). Some of these rule-based heuristics and ‘if-then’ procedures might be exploited while making decisions in dynamic tasks (Jenkins et al., 2011). But decision-making usually involves dynamic environments that rapidly change as a function of the feedback received from an individual (Gonzalez et al., 2005). It is understood that through proper training and skill acquisition, individuals can get more accustomed to the environment and the causal relationships that exist within the environment (Dreyfus, 2014). This, in turn, would enable the individuals to adapt to uncertainty and develop the adroitness and tactics for efficient decision-making in the face of unexpected events (Klein and Hoffman, 1993).

Many training methods exist for delivering enhanced performance in complex and dynamic systems. For instance, Salas et al. (2006) classified the existing training methods into information-based training methods, demonstration-based training methods, and practice-based training methods. According to Dreyfus and Dreyfus (1986), the information-based and demonstration-based training methods impart generalized training independent of context. For training dynamic decision-making, more experiential and naturalistic training might be required (Jenkins et al., 2011) so that individuals learn to make decisions at a more knowledge-based level. However, these naturalistic conditions’ design and development might be expensive, time-consuming, and dangerous in real-world settings (Stanton, 1996). Due to the dynamism associated with naturalistic environments, it becomes challenging for individuals to relate actions with ramifications (Jenkins et al., 2011). This makes it very difficult for individuals to develop rule-based heuristics and decision-making skills for a specific operation (Jenkins et al., 2011). This statement is particularly true in dynamic decision-making in the military context, where the conditions are usually unforeseeable and stressful (Stanton, 1996).

Research in recent years has proposed synthetic virtual environments as a more viable and reliable replacement to standard physical environments (Jenkins et al., 2011). These virtual environments provide highly customizable, flexible, and inexpensive training platforms for individuals to hone their decision-making skills (Naikar and Saunders, 2003; Jenkins et al., 2011). These virtual environments have traditionally been designed to be projected on a non-immersive computer screen (ter Haar, 2005), and more recently, in virtual reality (VR)/augmented reality (AR) using a head-mounted display (HMD) or on a cave automatic virtual environment (CAVE) (Srivastava et al., 2019). Of these, virtual environments in HMD VR have commonly been studied by researchers to assess and enhance the cognitive performance of individuals (Srivastava et al., 2019). In contrast to traditional non-immersive computer screens, VR allows individuals to immerse themselves in a virtual environment, move freely and seamlessly in the virtual environment, and examine the constituents governing the environment from all possible perspectives (ter Haar, 2005). As explained by Srivastava et al. (2019), HMD VR enables the participant to completely disconnect from the real physical environment around them by blocking eye contact and providing self-motion feedback. This feedback allows the individual to foster strategies they would employ in the real world in the virtual environment (Srivastava et al., 2019). As a result, this allows the individual to build a better mental model of the environment, leading to efficient cognitive skill acquisition (ter Haar, 2005).

Previous studies have illustrated the advantages of training individuals in HMD VR compared with non-immersive virtual environments in desktop screens in spatial learning and motor tasks (Murcia-López and Steed, 2016; Parmar et al., 2016; Srivastava et al., 2019). These studies have highlighted the ability of HMD VR to effectively localize spatial information in the environment yielding better learning rates and higher accuracies. But these studies also pointed out the increased cognitive workload rates in immersive HMD VR compared with non-immersive desktop screens (Parmar et al., 2016). For example, Parmar et al. (2016) attributed the high cognitive workload rates in immersive HMD VR to higher information-processing requirements due to an influx of higher knowledge-based heuristics in the environment. In hindsight, Parmar et al. (2016) also pointed out that individuals might regulate and even control the cognitive workload and enhance their cognitive performance if they undertook repetitive training in immersive VR. Also, Murcia-López and Steed (2016) indicated that repetitive training in immersive VR might lead to a better understanding of the information processing intricacies required to successfully execute the objectives in each environment, potentially leading to better workload management and enhanced cognitive performance. However, a detailed evaluation of how cognitive workload can be decreased and dynamic decision-making performance can be increased in complex environments through different repetitive training frameworks is lacking and much needed in the literature. Besides, very little research exists on how the equilibrium between high cognitive processing demands and cognitive performance can be achieved in a dynamic decision-making environment.

Over the years, researchers have evaluated the efficacy of different training conditions on generic cognitive and motor tasks. These training conditions include the “desirable difficulty framework” (Bjork, 1994), the “retrieval effort hypothesis” (Pyc and Rawson, 2009), the “procedural reinstatement hypothesis” (Lohse and Healy, 2012), the “cognitive antidote hypothesis” (Chapman et al., 2016), and the “heterogeneity of practice hypothesis” (Gonzalez and Madhavan, 2011). Popular theories of decision-making could delineate the effectiveness of these training conditions. For example, the instance-based learning theory [IBLT] (Gonzalez and Dutt, 2011; Dutt and Gonzalez, 2012; Lejarraga et al., 2012), a theory of how individuals make decisions from experience, has described decision-making (especially in a dynamic context) very efficiently. According to IBLT, decision-making is a five-step process: recognition of the situation, judgment based on experience, choices among options based upon judgments, execution of the chosen actions, and the feedback to those experiences that leads to the chosen actions (Gonzalez and Dutt, 2011; Lejarraga et al., 2012). But most of the training conditions mentioned above have only so far been tested on generic cognitive or motor tasks, where the acquisition of mere rule-based heuristics would suffice for successfully executing the task. Researchers, namely Rao et al. (2018a,b, 2020a, 2022) have demonstrated that VR training would maximize performance if individuals are first trained in a difficult environment compared with an easy environment in a dynamic decision-making context. But these studies have only given a glimpse of what the combination of immersive HMD VR and different training conditions have to offer in enhancing dynamic decision-making and regulating cognitive workload. A comprehensive evaluation of repetitive training conditions and how these conditions could facilitate enhanced cognitive performance in immersive VR is needed in the literature.

The primary objective of this research is to test these expectations in an experiment with human participants where the type of training condition (heterogenous, difficult, and sham) is varied between subjects. As per IBLT, we hypothesize that the heterogenous training condition, owing to the succinct variations in the environment, would enable the decision-maker to store and retrieve salient instances (experiences) from the environment compared with those stored in difficult training conditions and the sham condition. To the best of the author’s knowledge, this work’s contribution is novel because this would be the first study to explore the influence of different training conditions in a dynamic decision-making context on human performance when given repeatedly.

In what follows, first, we provide an overview of the research involving immersive HMD VR environments and their huge potential of training individuals for enhanced cognitive performance. We also investigate the different instances where different training conditions have been used to improve performance on several cognitive and psychological tasks. Next, we describe an experiment to investigate training individuals’ performance and cognitive implications under three different training conditions (heterogenous, difficult, and sham) in immersive HMD VR. Finally, we give a detailed explanation of the results obtained and discuss the implications of our results in the real-world.

## Background

A considerable amount of research has been conducted on evaluating the benefits and setbacks of using a VR-based system compared with traditional desktop-based systems. For instance, Santos et al. (2008) evaluated the efficacy of HMD-based VR systems compared with conventional desktop-based systems in a navigation scenario. The results indicated that users performed significantly better with the desktop setup compared with the HMD-based VR system. However, the subjects reported that the HMD-based VR system is more intuitive and natural.

Eventually, researchers indicated that with the growth of technology, HMD-based VR systems could be a better alternative for training personnel and emotional regulation. For example, Chirico et al. (2016) evaluated the efficacy of HMD-based VR systems as a mood induction tool for engendering awe. The researchers discovered that since VR provided a sense of “presence,” i.e., bestowing each individual with the illusion of “being there,” it facilitated a more efficient assessment of the emotional experience (Chirico et al., 2016). They also reasoned that since VR also had the ability to provide both monocular depth cues and binocular disparity, the participants could empathize with various theoretical aspects of the awe programmed in the environment (Chirico et al., 2016).

Similarly, another study investigated the efficacy of HMD-based VR systems in aiding information recall (Krokos et al., 2019). Forty participants were divided equally and randomly into two between-subject conditions: HMD with head-tracking and traditional desktop systems with mouse-based interaction. Results suggested that information recall in the HMD with the head-tracking condition was significantly more accurate and quicker than that in the traditional desktop system (Krokos et al., 2019). The researchers reasoned that this was due to the innate ability of the HMD-based VR system to provide the participants with better spatial awareness, which in turn influenced the participants’ vestibular and proprioceptive senses (Krokos et al., 2019).

A few researchers have cast their aspersions and reservations on the efficacy of HMD-based VR systems in regulating workload and facilitating the natural extension of cognitive capacities, especially capacities related to spatial navigation. For instance, Srivastava et al. (2019) examined the effectiveness of HMD-based VR displays with conventional desktop-based systems on spatial learning. They found that the participants spent more time and perceived lesser motion sickness in the desktop-based system than that in the HMD-based VR system. They reasoned that the high motion sickness nullified the high visual fidelity of HMD-based VR and the cognitive workload induced by the system (Srivastava et al., 2019). But these researchers have restricted ambulatory locomotion in the HMD-based VR system in the study, causing a severe dent in the effectiveness of the HMD-based VR system in engaging the participant’s proprioceptive abilities.

The usability and the propensity of VR systems to elicit an emotional response from the participants have been well documented by Pallavicini et al. (2019). In this research, 24 participants played the popular commercial game “Smash Hit” both in immersive (VR) and non-immersive (desktop-based) conditions. They acquired data related to the usability (through the system usability score questionnaire), the presence (through the Slater-Usoh-Steed presence questionnaire), and obtained objective responses from the participants during gameplay from physiological measures like heart rate and skin conductance response (Pallavicini et al., 2019). The results revealed that even though there is no significant difference between the VR and the desktop-based conditions in usability and performance, participants in the VR condition reported a higher sense of presence, and higher perceived happiness and surprise. In addition, Wang et al. (2018) evaluated the effect of HMD-based VRs compared with conventional desktop-based interfaces on the user’s sense of presence and self-efficacy in an educational context. Results revealed that the participants reported a higher sense of presence and recorded higher self-efficacy scores in the HMD-based VR condition compared with the conventional desktop-based condition (Wang et al., 2018).

The usability of VR [and as an extension, extended reality (ER) systems in general] in an effective and efficient emotional state detection has been well documented by Antoniou et al. (2020). In this research, 11 participants across diverse educational levels (spanning across undergraduate neurosurgeons, postgraduate neurosurgeons, and specialist neurosurgeons) wore an array of physiological sensors (for heart rate, electrodermal response, and electroencephalography recording) while executing two tasks in the Microsoft HoloLens VR/MR platform. Results indicated that all the physiological sensors are successfully able to detect discriminatory changes in the emotional state in the two tasks across the different groups of participants. Recently, state-of-the-art deep learning techniques have also been applied for accurate emotion detection in an immersive VR environment using electroencephalography (Ding et al., 2020).

Over the years, substantial research has been conducted on testing the efficacy of different repetitive training methods in basic cognitive processes. For instance, Bjork (1994) has proposed the ‘desirable difficulty framework’ for effective learning, which stated that within any learning domain, the difficult but successful processing of memory would lead to a better transfer compared with difficult but unsuccessful processing. Delving into the desirable difficulty framework, Pyc and Rawson (2009) proposed the ‘retrieval effort hypothesis’ which further substantiated Bjork’s (1994) claims to discover that difficult but successful retrievals lead to a better transfer compared with easier but successful retrievals. To test the retrieval effort hypothesis, they set up an experiment involving a Swahili-English translation task with conditions where retrieval during the practice trials is successful but disparately difficult. Results indicated that the performance level in the test condition increased as the difficulty of retrieval in the practice condition increased (Pyc and Rawson, 2009).

In addition, Lohse and Healy (2012) argued that training individuals in procedural information lead to better retention of “if-then’ procedures and rule-based heuristics than training individuals on declarative information. But they also found out that declarative information led to more robust and efficient information transfer than procedural information. Also, Chapman et al. (2016) determined that the addition of cognitively demanding stimuli to generic tasks leads to an increase in the accuracy and efficiency of the primary task being executed by the individual. Furthermore, Gonzalez and Madhavan (2011) ascertained that diversity and variation of stimuli during training lead to enhanced performance during the transfer. To test this hypothesis, the participants were trained in a luggage screening task in three different conditions: high diversity (higher number of categories of objects), low diversity (lower number of categories of objects), and sham condition, where the participants were not given any training (Gonzalez and Madhavan, 2011). After executing the training condition, the participants executed a test condition where they were instructed to look for novel objects in the screening task. Results indicated that the participants in the high diversity condition recorded significantly higher hit rates and faster response times in the test condition compared with the low diversity and the sham condition (Gonzalez and Madhavan, 2011).

However, the efficacy of these training methods has not been proven in complex and dynamic environments. Complex and dynamic environments usually demand a better encapsulation of skills, knowledge, and rules as per Rasmussen (1983). A comprehensive evaluation of different training methods in a naturalistic, dynamic decision-making environment is lacking and much needed in the literature. In this research work, we intend to address this gap in the literature by conducting an experiment to evaluate the efficacy of different repetitive training frameworks (heterogeneity and difficulty) in immersive VR in a complex search-and-shoot environment.

## Materials and methods

### Participants

Sixty-four participants (42 men and 22 women; mean age = 22.9 years, SD = 1.79 years) at the Indian Institute of Technology Mandi, Himachal Pradesh, India, the Department of Biomedical Engineering, Institute of Nuclear Medicine and Allied Sciences, and Defence Research and Development Organization, Delhi, India took part in this study. The experiment was approved by an institutional Ethical Committee at the Indian Institute of Technology Mandi and the Institute of Nuclear Medicine and Allied Sciences (IITM/DRDO-LSRB/VD/301). Since the recruitment of subjects was done through the STEM student pool available at two reputed universities, we were certain that none of the subjects had any military background. The recruitment for the study was done through formal announcements during class/office hours regarding the experiment. As a part of the demographics data acquired, the participants self-reported that they seldom played any games on any platform, i.e., less than 30 min of gaming per month on mobile phones, desktops, PlayStation, etc. In addition, the participants also self-reported that they had not experienced virtual reality in any shape or form before. We also included questions on the possibility of participants having frequent migraine-induced headaches and nausea, so the participants who reported the same would be excluded before beginning the experiment. This was because some researchers (de Tommaso et al., 2013; Vekhter et al., 2020) had suggested excluding participants with nausea and migraine because of possible exacerbation during the longitudinal VR intervention. All the participants gave a written consent form before they took part in the experiment. All the participants were from Science, Technology, Engineering, or Mathematics background. None of the participants reported any history of mental/psychiatric/neurological disorders. All the participants had normal or corrected-to-normal vision. Out of the 64 participants, 60 were right-handed. All the participants reported that they had never experienced virtual reality before. All the participants received a flat payment of INR 100 for their participation in the study.

### The dynamic decision-making simulation

**FIGURE 1:**
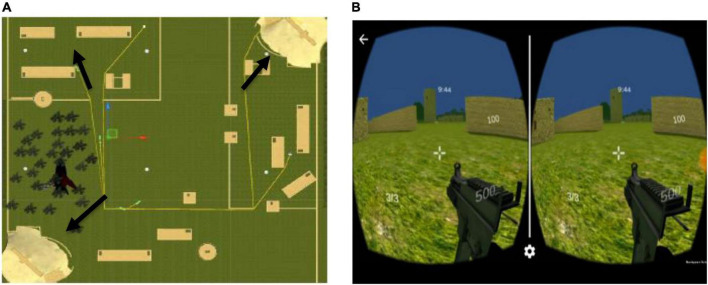
**(A)** Overhead map of the terrain-based search-and-shoot environment designed in Unity3D. The arrows indicate the locations of the army bases sieged by the enemies. **(B)** The HMD VR interface, indicating the time remaining at the top, the participant’s health at the middle-right corner, the number of army bases to be required from the enemies in the bottom-left corner and the number of bullets remaining at the bottom-right corner of the interface. Reproduced with permission from Rao et al. (2020b).

**FIGURE 2:**
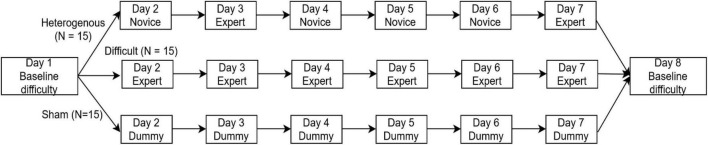
Experiment Design of the study. Participants performed in a VR environment of Baseline difficulty on Day 1 and were equally and randomly divided into three between-subjects conditions: Heterogenous, Difficult, and Sham. Each between-subjects condition was 6 days long (from Days 2 to 7) and consisted of Novice, Expert, and Dummy VR environments. On Day 8, all participants were again asked to perform in a VR environment of Baseline difficulty.

A search-and-shoot simulation was designed for android head mounted display (HMD) VR using Unity3D version 5.5 (Wang et al., 2010). The 3D avatars of the enemies in the simulation were created using Blender Animation version 2.79a (Flavell, 2011). As shown in Figure 1A, the simulation comprised three army bases located at different sites in the simulation (Rao et al., 2020a). Before the subjects began the task, it was explained to the participants that the enemies in the simulation had acquired these three army bases and the main objective of the participant was to kill all the enemies in the simulation and reacquire all the army bases in 10 min. The participant’s health in the simulation was initialized to 100 and this health would decrease based on the difficulty level of the simulation (Rao et al., 2020a). The total number of enemies in the simulation was 15. Three different levels of task difficulty (novice, expert, and baseline) were created for the search-and-shoot VR simulation. The difficulty levels were imbibed by making several characteristic changes in the simulation dynamics and incorporating behavior/AI-based changes on the enemy avatars (Rao et al., 2020a). A detailed explanation of the different levels of difficulty, incorporated in the simulation, is given in section “Variation in task difficulty.” As shown in Figure 1B, the 3D VR simulation was executed using an android smartphone (Xiaomi Redmi Note 3) and a MyVR HMD at a field-of-view of 100 degrees. The participant used a DOMO Magickey Bluetooth controller to maneuver the player avatar and shoot in the VR simulation. As shown in Figure 2, the participant was erect while executing the simulation. The experiment was conducted in an isolated laboratory.

### Experiment design

Figure 2 shows the experimental design followed. As shown in Figure 2, the subjects were equally and randomly divided into three between-subject conditions: heterogenous, difficult, and sham. The experiment was 8 days long. The subjects in the heterogenous condition executed the VR environment in baseline difficulty on Day 1. From Days 2 to 7, they alternatively executed the novice difficulty and expert difficulty versions of the environment, i.e., environment with novice difficulty on Day 2, environment with expert difficulty on Day 3, etc. On Day 8, they executed the environment with baseline difficulty. The baseline version of the VR environment executed by the participants on Day 1 and Day 8 was an amalgamation of the novice and expert versions of the environment. A detailed description of the variations in the difficulty introduced in the VR environment is given in section “Variation in task difficulty.” The subjects in the difficult condition executed the environment with baseline difficulty on Day 1. From Days 2 to 7, they executed the environment with expert difficulty and then executed the environment with baseline difficulty on Day 8. The subjects in the sham condition executed the environment with baseline difficulty on Day 1. From Days 2 to 7, the participants in the sham condition underwent VR training in a dummy scenario irrelevant to the search-and-shoot environment. This dummy environment was not related to the VR task executed by the subjects in the heterogenous and difficult conditions in any way. This dummy scenario was a VR simulation readily available for Android, called VR thrills: Roller Coaster 360 (Cardboard Game) (McIlroy et al., 2016). On Day 8, they again executed the VR environment with baseline difficulty.

On the first day, all the participants were briefed about the experiment, the VR environment, and the objectives to be achieved in the VR environment. The participants then filled out a consent form approving their voluntary participation in the experiment. The participants then filled out a demographic form consisting of questions pertaining to their age, gender, educational background, dominant hand, psychological/neurological history, possible nausea/oculomotor sickness, and virtual reality experience, among others. Then, all the participants executed a 10-min habituation session in a dummy VR environment to get acclimatized to the level of immersion, locomotion, and shooting inside an immersive virtual environment. Various performance measures like percentage of enemies killed (calculated by dividing the number of enemies killed by the participant/total number of enemies, i.e., 15, then multiplying it by 100), total time taken to complete the task, accuracy index (calculated by dividing the number of bullets taken by the participant to kill the enemies/number of bullets needed to kill the enemies, then multiplying it by 100), and health index (calculated by initial health – final health/time taken). The percentage of enemies killed and the accuracy index were performance measures that indicated higher attentional acuity and processing speed (Boot et al., 2008). The time taken and the health index were performance measures that reflected sustained vigilance and resilience (Jenkins et al., 2011). The performance measures were acquired after the task completion on all days. In addition, participants also undertook a computerized version of the NASA-task load index (TLX) (Hart, 2006). The NASA-TLX is a widely used scale for measuring participants’ perceived workload on a 10-point Likert scale (Hart, 2006), usually after the execution of tasks requiring considerable workload exertion. The NASA-TLX is divided into six sub-scales: mental demand, physical demand, temporal demand, performance satisfaction, frustration level, and effort. The participants filled out the NASA-TLX after the execution of the environments on Day 1 and Day 8. We carried out one-way ANOVAs to evaluate the main effect of the different types of training conditions (heterogenous, difficult, and sham) on various performance and cognitive measures. We also carried out one-way ANOVAs to evaluate the main effect of the duration of training (i.e., Day 1 and Day 8) on various performance and cognitive measures. In addition, we also carried out mixed ANOVAs to evaluate the interaction effects across different training conditions (heterogenous, difficult, and sham) and duration of training (Day 1 and Day 8) on the performance and cognitive measures. We also carried out a mixed factorial ANOVA to evaluate the interaction effects across different types of training conditions (heterogenous, difficult, and sham) and the entire duration of training (from Days 1 to 8) on the various performance measures. The Q-Q plots, i.e., the plot between expected and normal quantiles showed that all the dependent variables taken into consideration were normally distributed. The alpha level was set at 0.05 and the power was set at 0.8. All the statistical analysis in the article was done using IBM SPSS Statistics 20. We have strictly adhered to the methods cited by Field (2009) for executing all the statistical methods mentioned in the article. For executing the mixed factorial ANOVA, methods mentioned by Field (2009) (pg. no. 430–441) were strictly followed. For executing the repeated measures ANOVA, methods mentioned by Field (2009) (pg. no. 468–481) were strictly followed. The experimental data were then checked by a statistician at the University for accuracy. Overall, on account of IBLT (Gonzalez and Dutt, 2011; Dutt and Gonzalez, 2012; Lejarraga et al., 2012), heterogeneity of practice hypothesis (Gonzalez and Madhavan, 2011), desirable difficulty framework (Bjork, 1994), and retrieval effort hypothesis (Pyc and Rawson, 2009), we expected the participants to perform better when heterogenous and difficult training was imparted compared with the sham condition. In addition, on account of IBLT (Gonzalez and Dutt, 2011; Dutt and Gonzalez, 2012; Lejarraga et al., 2012) and the heterogeneity of practice hypothesis (Gonzalez and Madhavan, 2011), we expected the participants to perform better on Day 8 when the heterogenous training condition was imparted compared with the sham condition.

### Variation in task difficulty

The variation in the characteristic attributes of the simulation and the enemy avatar concerning the type of task difficulty is shown in Table 1. As shown in Table 1, the ammunition available for the participants in all the difficulty levels was constant at 500. The delay between successive shots by the enemy was kept to the following: 30 frames in the novice difficulty level and 15 frames in the expert and baseline difficulty levels (Rao et al., 2020a). The rate of health decrease of the enemy per shot was kept to the following: 10 in the novice and baseline difficulty levels and 8 in the expert difficulty level. The rate of health decrease of the player per shot was kept to the following: 1 in the novice difficulty level and 2 in the expert and baseline difficulty levels (Rao et al., 2020a). The field-of-view of the enemy was kept to the following: 90° in the novice and the baseline difficulty levels and 120° in the expert difficulty level (Rao et al., 2020a). The total time available to complete the objectives in the VR environment was kept to 10 min in all the difficulty levels.

**TABLE 1:** Variation in the physical attributes of the environment with respect to the different difficulty levels.

Attribute	Novice difficulty level	Expert difficulty level	Baseline difficulty level
Ammunition	500	500	500
Delay between consecutive shots by the enemy avatar	30 frames	15 frames	15 frames
Rate of decrease in health of the enemy avatar per shot	10	8	10
Rate of decrease in health decrease of the player avatar per shot	1	2	2
FoV of the enemy avatar	90°	120°	90°
Total time to complete the simulation	10 min	10 min	10 min

**FIGURE 3:**
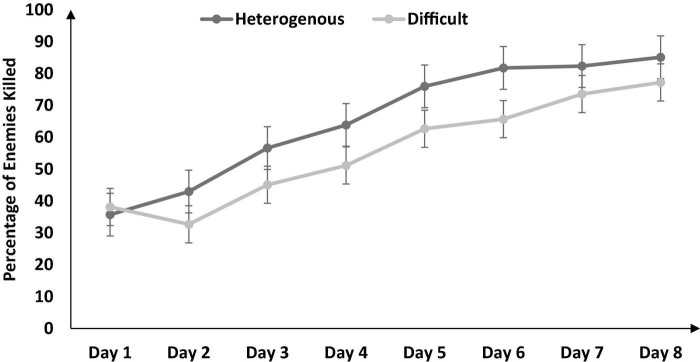
The percentage of enemies killed across different training conditions and days. The error bars show the 95% CI around point estimates.

The physical mesh was divaricated into three sections: fully covered areas (in light blue), partially open areas (in purple), and open areas (in green). As shown in Figure 3, the areas covered in light blue constitute areas with hiding spots for the enemy avatars and the participant. The purple areas depicted the areas with a medium number of hiding spots and the green areas depicted the fully open areas with no hiding spots whatsoever (Rao et al., 2020a).

We used finite state machines (FSMs) to ascertain the actions associated with the behavior of the enemies in the simulation (Rao et al., 2020a). The enemies were divided into two coteries: three assault groups of enemies and three stealth enemies. Each assault group consisted of four enemies where one was the leader and the other three were followers. The leader would decide the behavior/movement to be executed and the followers would follow suit. The assault groups of enemies were indifferent to the cost of the areas and were programmed to move to four possible destinations – randomly, toward the participant, toward an army base, or stay at the same location (Rao et al., 2020a). The probabilities of the assault group and the stealth group moving to all these destinations were varied according to the difficulty levels (see Table 2). As shown in Table 2, the probability of the assault group moving toward the participant was kept to the following: 0.25 in the novice difficulty level and 0.75 in the expert and baseline difficulty levels. The probability of the assault group moving toward the army base was kept to the following: 0.25 in the novice and baseline difficulty levels and 0.75 in the expert difficulty level (Rao et al., 2020a). The probability of the assault group moving randomly was kept to the following: 0.75 in the novice and baseline difficulty levels and 0.25 in the expert difficulty level. The probability of the assault group remaining static was kept to the following: 0.75 in the novice difficulty level and 0.25 in the expert and baseline difficulty levels. The stealth group, which consisted of three enemy avatars, acted independently to the assault enemies. The stealth group walked in areas of the physical mesh with minimum cost. As shown in Table 2, the probability of the stealth group moving toward an army base was kept to the following: 0.3 in the novice difficulty level and 0.7 in the expert and baseline difficulty levels. The probability of the stealth group moving randomly was kept to the following: 0.7 in the novice and the baseline difficulty levels and 0.3 in the expert difficulty level (Rao et al., 2020a).

**TABLE 2:** Variation in probabilities of movement with respect to different difficulty levels.

Attribute	Novice difficulty level	Expert difficulty level	Baseline difficulty level
Toward participant (assault)	0.25	0.75	0.75
Toward army base (assault)	0.25	0.75	0.25
Random (assault)	0.75	0.25	0.75
Static (assault)	0.75	0.25	0.25
Toward army base (stealth)	0.30	0.70	0.70
Random (stealth)	0.70	0.30	0.70

## Results

### Performance measures

#### Percentage of enemies killed

**FIGURE 4:**
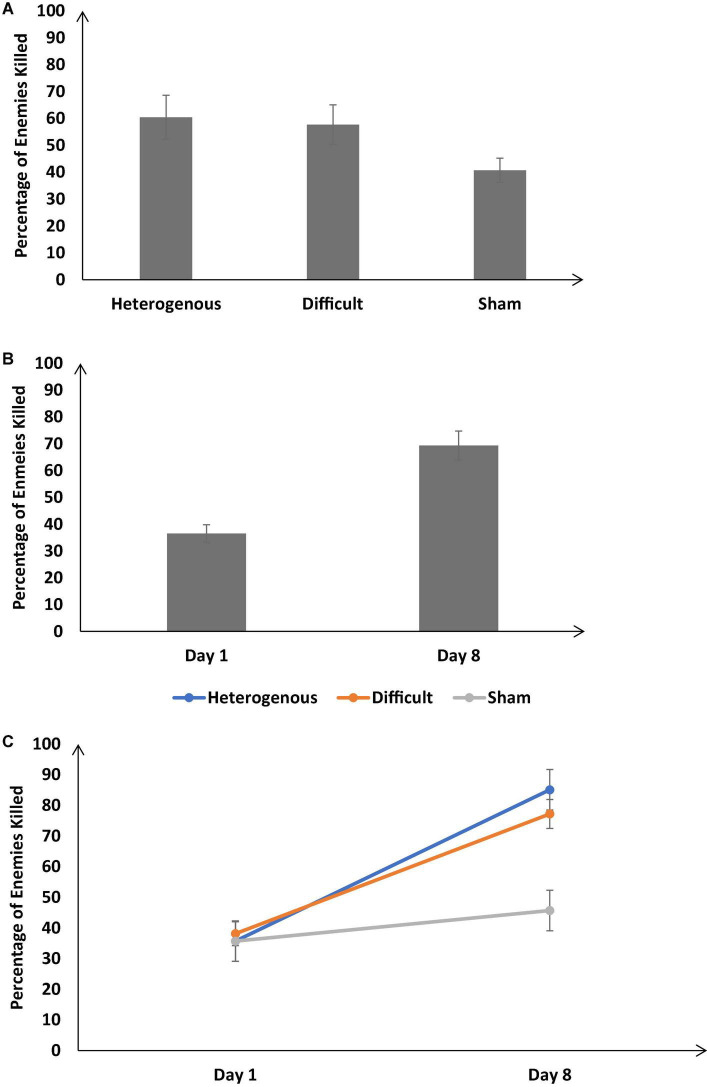
**(A)** Percentage of enemies killed across different training conditions. **(B)** Percentage of enemies killed across Day 1 and Day 8. **(C)** Percentage of enemies killed across different training conditions and Day 1 and Day 8. The error bars show 95% CI around point estimates.

Figure 4A shows the percentage of enemies killed across the different training conditions. As shown in Figure 4A, the percentage of enemies killed was significantly different across different training conditions [*F* (2, 126) = 25.57, *p* < 0.05, η_*p*_^2^ = 0.28]. The Bonferroni *post hoc* test revealed the percentage of enemies killed was similar in the heterogenous and difficult conditions; however, there was a significant difference between the heterogenous and sham conditions and difficult and sham conditions [Heterogenous: μ = 60.41% ∼ Difficult: μ = 57.68% (*p* = 0.9), Heterogenous: μ = 60.41% > Sham: μ = 40.72% (*p* < 0.05); Difficult: μ = 57.68% > Sham: μ = 40.72% (*p* < 0.05)].

**FIGURE 5:**
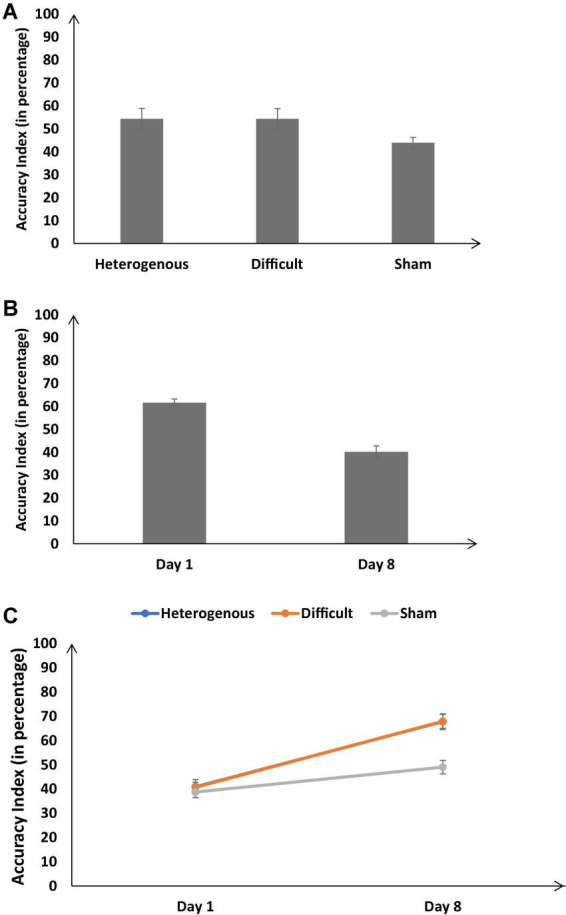
**(A)** The accuracy index (in percentage) across different training conditions. **(B)** The accuracy index (in percentage) on Day 1 and Day 8. **(C)** The accuracy index (in percentage) across different training conditions on Day 1 and Day 8. The error bars show 95% CI around point estimates.

Figure 4B shows the percentage of enemies killed on Day 1 and Day 8 across all training conditions. As shown in Figure 5B, across all conditions, the percentage of enemies killed was significantly higher on Day 8 compared with Day 1 [Day 8: μ = 69.34% > Day 1: μ = 36.52%; *F* (1, 126) = 89.84, *p* < 0.05, η_*p*_^2^ = 0.59].

Furthermore, the interaction between the type of training and the duration of training significantly influenced the percentage of enemies killed [*F* (2, 126) = 23.16, *p* < 0.05, η_*p*_^2^ = 0.26] (See Figure 4C). The Bonferroni *post hoc* test revealed that there was no difference in the percentage of enemies killed between different conditions on Day 1 [Heterogenous: μ = 35.72% ∼ Difficult: μ = 38.14% (*p* = 0.9), Heterogenous: μ = 35.72% ∼ Sham: μ = 35.72% (*p* = 1); Difficult: μ = 38.14% ∼ Sham: μ = 35.72% (*p* = 0.9)]. However, the percentage of enemies killed was significantly higher in heterogenous and difficult conditions compared with the sham condition on Day 8 [Heterogenous: μ = 85.11% ∼ Difficult: μ = 77.21% (*p* = 0.06), Heterogenous: μ = 85.11% > Sham: μ = 45.70% (*p* < 0.05); Difficult: μ = 77.21% > Sham: μ = 45.70% (*p* < 0.05)]. Overall, as per our expectations, both the heterogenous and difficult training conditions yielded an increase in the percentage of enemies killed on Day 8 compared with the sham condition.

Next, we compared the percentage of enemies killed between the heterogenous and difficult conditions throughout training (from Days 1 to 8) *via* a mixed-factorial ANOVA. Figure 3 shows the percentage of enemies killed in the heterogenous and difficult conditions throughout the training. Mauchly’s test for the percentage of enemies killed indicated that the assumption of sphericity was violated [*χ^2^*(27) = 83.89, *p* < 0.05]. Therefore, multivariate tests were reported with Greenhouse-Geisser correction (ε = 0.64). Results showed that percentage of enemies killed was significantly affected by the type of training [*F* (4.48, 188.23) = 14.1, *p* < 0.05, η_*p*_^2^ = 0.77]. In addition, the interaction between the type of training and the duration of training on the percentage of enemies killed was also significant [*F* (4.48, 188.23) = 3.56, *p* < 0.05, η_*p*_^2^ = 0.07]. As shown in Figure 3, the percentage of enemies killed had a dip on Day 2 in the difficult condition and a dip on Day 3 in the heterogenous condition (across both conditions, the percentage of enemies killed increased over days after the initial dip in performance).

#### Time taken

**FIGURE 6:**
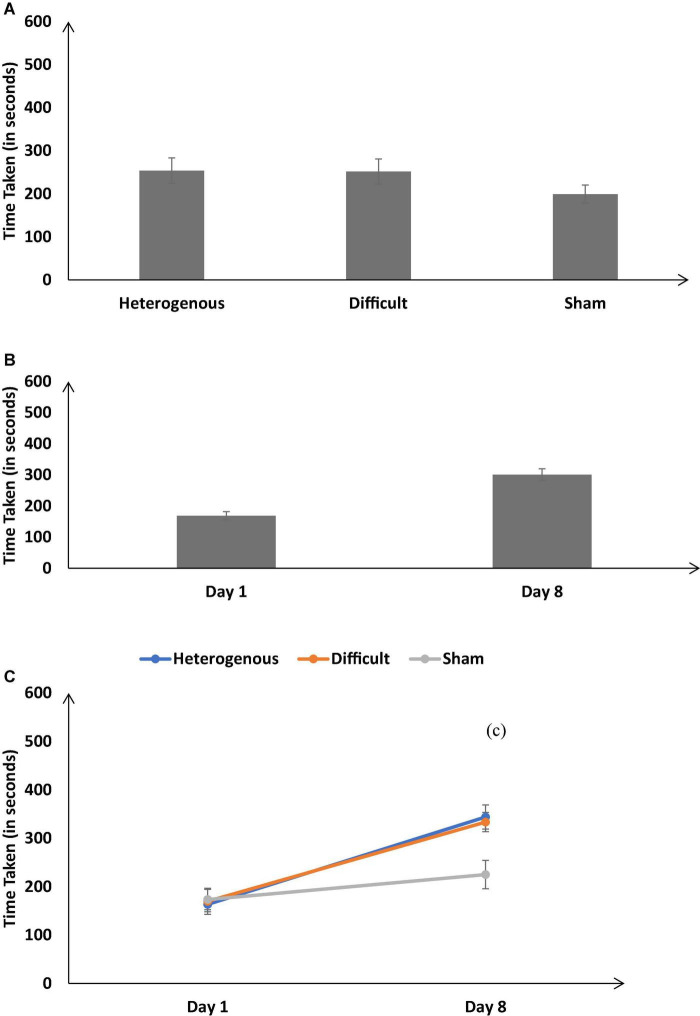
**(A)** The time taken (in seconds) across different training conditions. **(B)** The time taken (in seconds) on Day 1 and Day 8. **(C)** The time taken (in seconds) across different training conditions on Day 1 and Day 8. The error bars show 95% CI around point estimates.

The time taken to complete the simulation was not significantly different across different training conditions [*F* (2, 126) = 13.42, *p* < 0.05, η_*p*_^2^ = 0.18]. The Bonferroni *post hoc* test revealed the time taken was similar in the heterogenous and difficult conditions; however, there was a significant difference between the heterogenous and sham conditions and difficult and sham conditions [Heterogenous: μ = 253.63s ∼ Difficult: μ = 251.59s (*p* = 0.9), Heterogenous: μ = 253.63s > Sham: μ = 199.11s (*p* < 0.05); Difficult: μ = 251.59s > Sham: μ = 199.11s (*p* < 0.05) as shown in Figure 6A].

Figure 6B shows the time taken on Day 1 and Day 8 across all training conditions. As shown in Figure 6B, across all conditions, the time taken was significantly higher on Day 8 compared with Day 1 [Day 1: μ = 300.77s < Day 8: μ = 168.78s; *F* (1, 126) = 183.54, *p* < 0.05, η_*p*_^2^ = 0.59].

Furthermore, the interaction between the type of training and the duration of training significantly influenced the time taken [*F* (2, 126) = 17.26, *p* < 0.05, η_*p*_^2^ = 0.21] (See Figure 6C). The Bonferroni *post hoc* test revealed that there were no difference in the time taken between the heterogenous and difficult conditions on Day 1 [Heterogenous: μ = 163.31s ∼ Difficult: μ = 169.72s (*p* = 0.9)]. In addition, there was no significant difference in the time taken between the heterogenous and sham conditions and the difficult and sham conditions on Day 1 [Heterogenous: μ = 163.31s < Sham: μ = 173.31s (*p* = 0.9), Difficult: μ = 169.72s < Sham: μ = 173.31s (*p* = 0.9)]. However, the time taken was significantly higher in the heterogenous and difficult conditions compared with the sham condition on Day 8 [Heterogenous: μ = 343.95s > Sham: μ = 224.9s (*p* < 0.05); Difficult: μ = 333.45s > Sham: μ = 224.9s (*p* < 0.05)]. There was no significant difference in the time taken between the heterogenous and difficult conditions on Day 8 [Heterogenous: μ = 343.95s ∼ Difficult: μ = 333.45s (*p* = 0.98)]. Overall, as per our expectations, both the heterogenous and difficult conditions yielded an increase in the time taken on Day 8 compared with the sham condition.

Next, we compared the time taken between the heterogenous and difficult conditions over the duration of training (from Days 1 to 8) *via* a mixed-factorial ANOVA. Mauchly’s test for the time taken indicated that the assumption of sphericity was not violated [*χ^2^*(27) = 39.07, *p* = 0.06]. Therefore, multivariate tests were reported with sphericity assumed. Results showed that the time taken was not significantly affected by the type of training [*F* (7, 294) = 0.58, *p* = 0.76, η_*p*_^2^ = 0.01]. There was no interaction between the type of training conditions and duration of training on the time taken [*F* (7, 196) = 0.66, *p* = 0.75, η_*p*_^2^ = 0.01].

#### Accuracy index

Figure 5A shows the percentage accuracy index across the different training conditions. As shown in Figure 5A, the percentage accuracy index was significantly different across different training conditions [*F* (2, 126) = 36.71, *p* < 0.05, η_*p*_^2^ = 0.37]. The Bonferroni *post hoc* test revealed that the percentage accuracy index was higher in the heterogenous condition compared with the sham condition [Heterogenous: μ = 54.36% ∼ Sham: μ = 43.9% (*p* < 0.05)]; however, the percentage accuracy index was similar in the heterogenous and difficult conditions and significantly different across difficult and sham conditions [Heterogenous μ = 54.36% ∼ Difficult: μ = 54.31% (*p* = 0.9), Difficult: μ = 54.31% > Sham: μ = 43.9% (*p* < 0.05)].

Figure 5B shows the percentage accuracy index on Day 1 and Day 8 across all training conditions. As shown in Figure 5B, the accuracy index was significantly higher on Day 8 compared with Day 1 [Day 8: μ = 61.6% > Day 1: μ = 40.12%; *F* (1, 126) = 350.39, *p* < 0.05, η_*p*_^2^ = 0.74].

Furthermore, the interaction between the type of training and the duration of training significantly influenced the percentage accuracy index [*F* (2, 126) = 23.87, *p* < 0.05, η_*p*_^2^ = 0.28] (see Figure 5C). The Bonferroni *post hoc* test revealed that there was no difference in the percentage accuracy index between different conditions on Day 1 [Heterogenous: μ = 40.91% ∼ Difficult: μ = 40.68% (*p* = 0.9), Heterogenous: μ = 40.91% ∼ Sham: μ = 38.77% (*p* = 0.85), Difficult: μ = 40.68% ∼ Sham: μ = 38.77% (*p* = 0.9)]. However, the percentage accuracy index was significantly higher in the heterogenous and difficult conditions compared with the sham condition on Day 8 [Heterogenous: μ = 67.81% > Sham: μ = 49.04% (*p* < 0.05), Difficult: μ = 67.95% > Sham: μ = 49.04% (*p* < 0.05)]. The percentage accuracy index in the heterogenous and difficult conditions was similar [Heterogenous: μ = 67.81% ∼ Difficult: μ = 67.95% (*p* = 0.9)]. Overall, as per our expectations, both the heterogeneity and difficult conditions yielded an increase in the percentage accuracy index on Day 8 compared with the sham condition.

**FIGURE 7:**
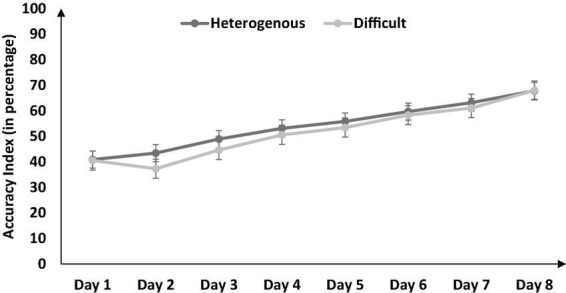
The accuracy index (in percentage) across different type of training conditions and baseline/training days (Day 1 to Day 8). The error bars show 95% CI around point estimates.

Next, we compared the percentage accuracy index between the heterogenous and difficult conditions over the duration of training (from Days 1 to 8) *via* a mixed-factorial ANOVA. Figure 7 shows the percentage accuracy index in the heterogenous and difficult conditions over the duration of training. Mauchly’s test for the accuracy index indicated that the assumption of sphericity was violated, [*χ^2^*(27) = 78.85, *p* < 0.05]. Therefore, multivariate tests were reported with Greenhouse-Geisser correction (ε = 0.675). Results showed that the percentage accuracy index was significantly affected by the type of training condition [*F* (4.72, 198.305) = 23.43, *p* < 0.05, η_*p*_^2^ = 0.73]. However, the interaction between the type of training and the duration of training on the percentage accuracy index was not significant [*F* (4.72, 198.31) = 1.29, *p* < 0.05, η_*p*_^2^ = 0.03]. In the heterogenous condition, the percentage accuracy index recorded a dip on Day 2 and then increased gradually till Day 8 (see Figure 7). In the difficult condition, the percentage accuracy index increased gradually till Day 7 and dipped a bit on Day 8.

#### Health index

The health index was not significantly different across different training conditions [*F* (2, 126) = 0.94, *p* = 0.53, η_*p*_^2^ = 0.04].

**FIGURE 8:**
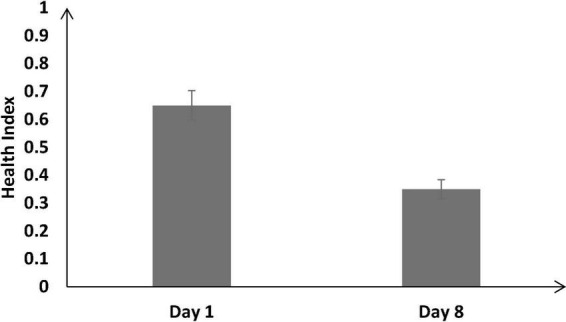
The health index across different baseline days (Day 1 and Day 8). The error bars show 95% CI around point estimates.

Figure 8 shows the health index on Day 1 and Day 8 across all training conditions. As shown in Figure 8, across all conditions, the health index was significantly lower on Day 8 compared with Day 1 [Day 8: μ = 0.35 < Day 1: μ = 0.65; *F* (1, 126) = 92.87, *p* < 0.05, η_*p*_^2^ = 0.42].

Furthermore, the interaction between the type of training and the duration of training did not significantly influence the health index [*F* (2, 126) = 2.56, *p* = 0.27, η_*p*_^2^ = 0.01].

Next, we compared the health index between the heterogenous and difficult conditions throughout the training (from Days 1 to 8) *via* a mixed-factorial ANOVA. Mauchly’s test for the health index indicated that the assumption of sphericity was violated, [*χ^2^*(27) = 84.32, *p* < 0.05]. Therefore, multivariate tests were reported with Greenhouse-Geisser correction (ε = 0.34). Results showed that the health index was not significantly affected by the type of training [*F* (1.45, 183.44) = 1.34, *p* = 0.32, η_*p*_^2^ = 0.03]. There was no significant interaction between the training conditions and the duration of training on the health index [*F* (1.45, 198.31) = 1.56, *p* = 0.45, η_*p*_^2^ = 0.02].

### Cognitive measures

#### Mental demand

**FIGURE 9:**
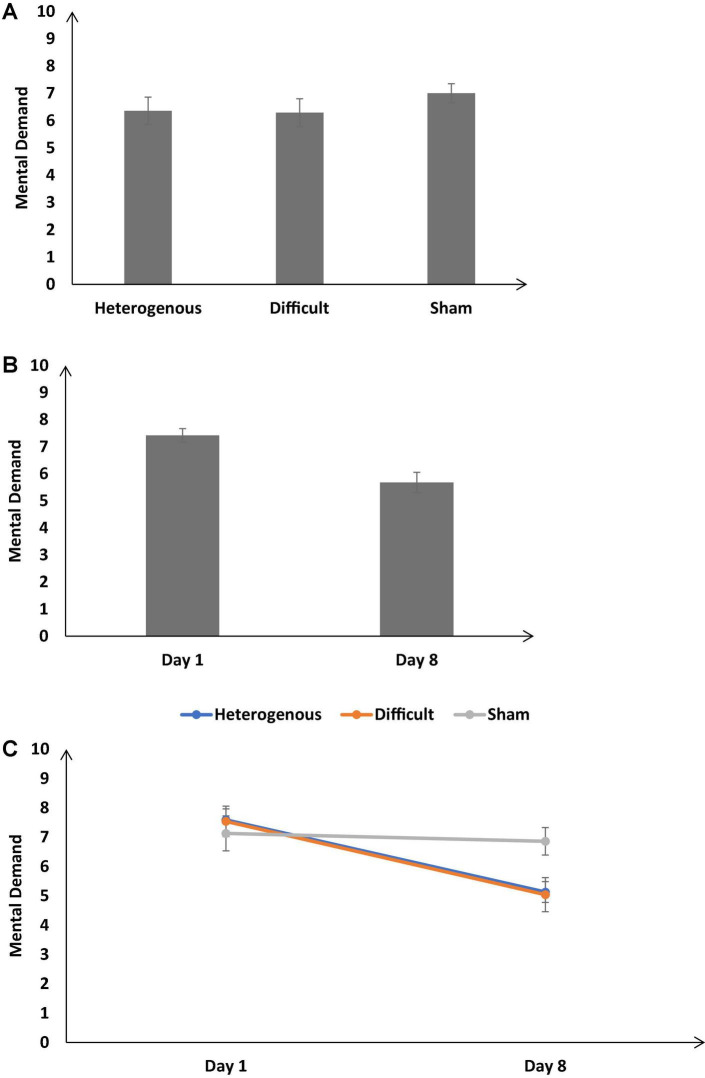
**(A)** The self-reported mental demand across different training conditions. **(B)** The mental demand across different baseline days (Day 1 and Day 8). **(C)** The mental demand across different training conditions and baseline days (Day 1 and Day 8). The error bars show 95% CI around point estimates.

Figure 9A shows the self-reported mental demand across the different training conditions. As shown in Figure 9A, the self-reported mental demand was significantly different across different training conditions [*F* (2, 126) = 17.82, *p* < 0.05, η_*p*_^2^ = 0.17]. The Bonferroni *post hoc* test revealed that the self-reported mental demand was significantly higher in the sham condition compared with the heterogenous condition and the sham condition compared with the difficult condition [Heterogenous: μ = 6.36 < Sham: μ = 7 (*p* < 0.05), Difficult: μ = 6.29 < Sham: μ = 7 (*p* < 0.05)]. The self-reported mental demand was similar across the heterogenous and difficult conditions [Heterogenous: μ = 6.36 ∼ Difficult: μ = 6.29 (*p* = 0.99)].

Figure 9B shows the self-reported mental demand on Day 1 and Day 8 across all conditions. As shown in Figure 9B, across all conditions, the mental demand was significantly lower on Day 8 compared with Day 1 [Day 8: μ = 5.68 < Day 1: μ = 7.42; *F* (1, 126) = 37.85, *p* < 0.05, η_*p*_^2^ = 0.36].

Furthermore, the interaction between the type of training and the duration of training significantly influenced the self-reported mental demand [*F* (2, 126) = 17.82, *p* < 0.05, η_*p*_^2^ = 0.17] (see Figure 9C). The Bonferonni *post hoc* test revealed that there was no difference in the self-reported mental demand between different conditions on Day 1 [Heterogenous: μ = 7.59 ∼ Difficult: μ = 7.54 (*p* = 0.9), Heterogenous: μ = 7.59 ∼ Sham: μ = 7.13 (*p* = 0.56), Difficult: μ = 7.54 ∼ Sham: μ = 7.13 (*p* = 0.61)]. However, the self-reported mental demand was significantly lower in heterogenous, and difficult conditions compared with the sham condition on Day 8 [Heterogenous: μ = 5.13 < Sham: μ = 6.86 (*p* < 0.05), Difficult: μ = 5.04 < Sham: μ = 6.86 (*p* < 0.05)]. The self-reported mental demand was similar across heterogenous and difficult conditions on Day 8 [Heterogenous: μ = 5.13 ∼ Difficult: μ = 5.04 (*p* = 0.9)]. Overall, as per our expectations, both the heterogenous and difficult training conditions yielded a decrease in the self-reported mental demand on Day 8 compared with the sham condition.

#### Physical demand

**FIGURE 10:**
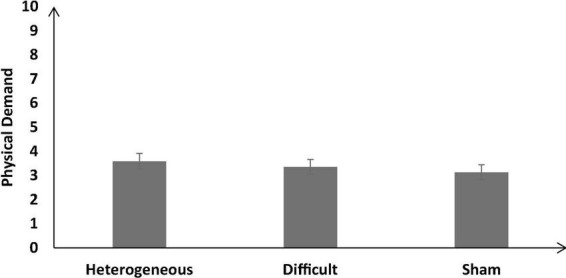
The self-reported physical demand across different training conditions. The error bars show 95% CI around point estimates.

Figure 10 shows the self-reported physical demand across the different training conditions. As shown in Figure 10, the self-reported physical demand was not significantly different across different training conditions [*F* (2, 126) = 2.27, *p* = 0.12, η_*p*_^2^ = 0.03]. The Bonferroni *post hoc* test revealed that the physical demand was similar in the heterogenous condition compared with the difficult condition [Heterogenous: μ = 3.59 ∼ Difficult: μ = 3.36 (*p* = 0.92)]. In addition, the self-reported physical demand was similar in the heterogenous condition compared with the sham condition [Heterogenous: μ = 3.59 > Sham: μ = 3.13 (*p* = 0.12)].

The self-reported physical demand did not yield any statistically significant difference across all training conditions when Day 8 was compared with Day 1 [Day 8: μ = 3.31 ∼ Day 1: μ = 3.4; *F* (1, 126) = 2.09, *p* = 0.13, η_*p*_^2^ = 0.03].

Furthermore, the interaction between the training type and the duration did not significantly influence the self-reported physical demand [*F* (2, 126) = 4.01, *p* = 0.06, η_*p*_^2^ = 0.06].

#### Temporal demand

The self-reported temporal demand was not significantly different across different training conditions [*F* (2, 126) = 1.18, *p* = 0.31, η_*p*_^2^ = 0.02].

Across all conditions, the self-reported temporal demand did not yield any statistical significance when Day 8 was compared with Day 1 [*F* (1, 126) = 0.62, *p* = 0.92, η_*p*_^2^ = 0.05].

Furthermore, the interaction between the training type and the duration did not significantly influence the self-reported temporal demand [*F* (2, 126) = 1.45, *p* = 0.24, η_*p*_^2^ = 0.02].

#### Frustration level

The self-reported frustration level was not significantly different across different training conditions [*F* (2, 126) = 2.05, *p* = 0.13, η_*p*_^2^ = 0.03].

Across all conditions, the self-reported frustration level did not yield any statistical significance when Day 8 was compared with Day 1 [Day 8: μ = 4.4 ∼ Day 1: μ = 4.33; *F* (1, 126) = 0.12, *p* = 0.73, η_*p*_^2^ = 0.001].

Furthermore, the interaction between the type of training and the duration of training did not significantly influence the self-reported frustration level [*F* (2, 126) = 2.05, *p* = 0.13, η_*p*_^2^ = 0.032].

#### Performance satisfaction

**FIGURE 11:**
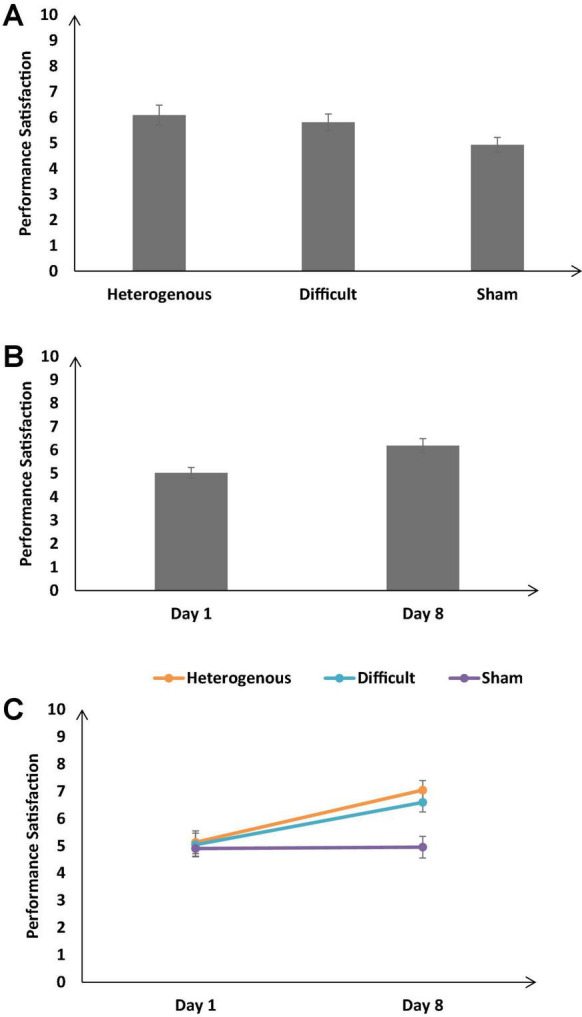
**(A)** The self-reported performance satisfaction across different training conditions. **(B)** The performance satisfaction across different baseline days (Day 1 and Day 8). **(C)** The performance satisfaction across different training conditions and baseline days (Day 1 and Day 8). The error bars show 95% CI around point estimates.

**FIGURE 12:**
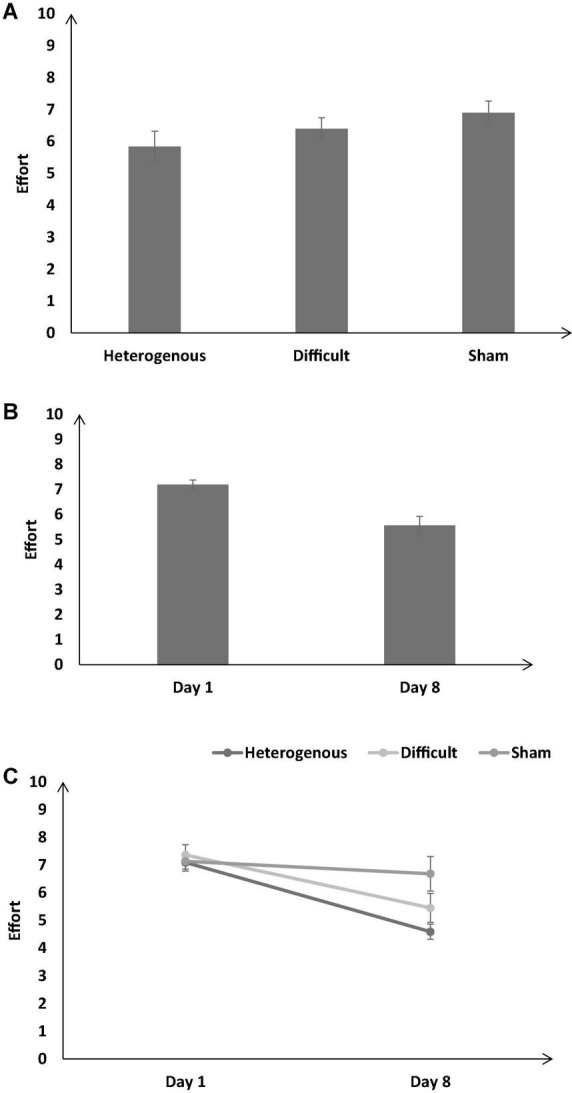
**(A)** The self-reported effort across different training conditions. **(B)** The effort across different baseline days (Day 1 and Day 8). **(C)** The effort across different training conditions and baseline days (Day 1 and Day 8). The error bars show 95% CI around point estimates.

Figure 11A shows the self-reported performance satisfaction across the different training conditions. As shown in Figure 12A, the self-reported performance satisfaction was significantly different across different training conditions [*F* (2, 126) = 13.18, *p* < 0.05, η_*p*_^2^ = 0.24]. The Bonferroni *post hoc* test revealed the self-reported performance satisfaction was significantly higher in the heterogenous condition compared with the sham condition and the difficult condition compared with the sham condition [Heterogeneity: μ = 6.09 > Sham: μ = 4.93 (*p* < 0.05), Difficult: μ = 5.81 > Sham: μ = 4.93 (*p* < 0.05)]. The Bonferroni *post hoc* test also revealed that the self-reported performance satisfaction was similar across the heterogenous and difficult training condition [Heterogeneity: μ = 6.09 ∼ Difficult: μ = 5.81 (*p* = 0.48)].

Figure 11B shows the self-reported performance satisfaction across all conditions on Day 1 and Day 8. As shown in Figure 12B, across all conditions, the self-reported performance satisfaction was significantly higher on Day 8 compared with Day 1 [Day 1: μ = 6.19 < Day 8: μ = 5.03; *F* (1, 126) = 55.16, *p* < 0.05, η_*p*_^2^ = 0.3].

Furthermore, the interaction between the type of training and the duration of training significantly influenced the self-reported performance satisfaction [*F* (2, 126) = 13.18, *p* < 0.05, η_*p*_^2^ = 0.17] (see Figure 11C). The Bonferroni *post hoc* test revealed that there was no significant difference in the self-reported performance satisfaction between different training conditions on Day 1 [Heterogenous: μ = 5.13 ∼ Difficult: μ = 5.04 (*p* = 0.9), Heterogenous: μ = 5.13 ∼ Sham: μ = 4.9 (*p* = 0.8), Difficult: μ = 5.04 ∼ Sham: μ = 4.9 (*p* = 0.9)]. However, the self-reported performance satisfaction was significantly higher in the heterogenous and difficult conditions compared with the sham condition on Day 8 [Heterogenous: μ = 7.04 > Sham: μ = 4.95 (*p* < 0.05), Difficult: μ = 6.59 > Sham: μ = 4.95 (*p* < 0.05)]. Overall, as per our expectations, both the heterogeneity and difficult training conditions yielded an increase in the self-reported performance satisfaction on Day 8 compared with the sham condition.

#### Effort

Figure 12A shows the self-reported effort across the different training conditions. As shown in Figure 12A, the self-reported effort was significantly different across different training conditions [*F* (2, 126) = 12.47, *p* < 0.05, η_*p*_^2^ = 0.16]. The Bonferroni *post hoc* test revealed that the self-reported effort was significantly higher in the sham condition compared with the heterogenous and difficult conditions [Heterogenous: μ = 5.84 < Difficult: μ = 6.4 (*p* < 0.05), Heterogenous: μ = 5.84 < Sham: μ = 6.9 (*p* < 0.05), Difficult: μ = 6.4 < Sham: μ = 6.9 (*p* < 0.05)].

Figure 12B shows the self-reported effort across all conditions on Day 1 and Day 8. As shown in Figure 12B, the self-reported effort was significantly lower on Day 8 compared with Day 1 [Day 1: μ = 7.19 > Day 8: μ = 5.57; *F* (1, 89) = 86.02, *p* < 0.05, η_*p*_^2^ = 0.4].

Furthermore, the interaction between the type of training and the duration of training significantly influenced the self-reported effort [*F* (2, 126) = 12.08, *p* < 0.05, η_*p*_^2^ = 0.16] (see Figure 12C). The Bonferroni *post hoc* test revealed that there was no significant difference in the self-reported effort between different conditions on Day 1 [Heterogenous: μ = 7.09 ∼ Difficult: μ = 7.36 (*p* = 0.75), Heterogenous: μ = 7.09 ∼ Sham: μ = 7.13 (*p* = 0.9), Difficult: μ = 7.36 ∼ Sham: μ = 7.13 (*p* = 0.6)]. However, the self-reported effort was significantly lower in the heterogenous and difficult conditions compared with the sham condition on Day 8 [Heterogenous: μ = 4.59 < Sham: μ = 6.68 (*p* < 0.05), Difficult: μ = 5.45 < Sham: μ = 6.68 (*p* < 0.05)]. The Bonferroni *post hoc* test also revealed that the self-reported effort was significantly lower in the heterogenous condition compared with the difficult condition on Day 8 [Heterogenous: μ = 4.59 < Difficult: μ = 5.45 (*p* < 0.05)]. Overall, as per our expectations, both the heterogenous and difficult training conditions yielded a decrease in the self-reported effort on Day 8 compared with the condition.

## Discussion and conclusion

This article investigated the efficacy of different repetitive training frameworks, i.e., heterogenous training and difficult training in immersive VR in a complex search-and-shoot scenario. Participants were divided into three between-subject conditions (heterogenous, difficult, and sham). All the participants executed a baseline version of the search-and-shoot environment on Day 1. The participants in the heterogenous condition alternatively undertook VR training in the novice and difficult versions of the search-and-shoot environment from Days 2 to 7. The participants in the difficult condition undertook VR training only in the difficult version of the search-and-shoot environment from Days 2 to 7. The participants in the sham condition underwent VR training in a dummy scenario irrelevant to the search-and-shoot environment. On Day 8, all the participants executed the baseline version of the VR search-and-shoot environment again. The complexity in the environment was varied by changing some basic physical characteristics of the enemy avatars appearing in the environment and by changing their AI. We took several performances and cognitive measures into consideration for our analysis and subsequent interpretation of results.

The advantages of using VR as the display medium for assessing and enhancing cognitive performance are well documented by Rao et al. (2018a, 2020a). In this study, VR allowed the possibility for the participant to immerse themselves in the virtual world, which eventually helped them to build a better mental model of the immediate environment. The advantage of VR as the display medium is evident in the significantly better performance obtained and significantly less mental demand reported by the participants to successfully execute the task. The immersion, the presence, i.e., the “sense of being there” (ter Haar, 2005) leads to a significantly more realistic engagement with the environment and its characters. This, coupled with the resolution offered by VR facilitated the creation of successful instances in the participant’s memory, leading to better information processing and performance. However, the extent of realism required in VR has been debated by some researchers (Gisbergen et al., 2019). Gisbergen et al. (2019) bought forth two contradictory theories on the extent of realism. One is Gestalt theory (Pertaub et al., 2002), which claims that high realism increases the experience and natural behavior. The other theory is called the Uncanny Valley (Slater and Steed, 2000), which states that too much realism and resemblance to the real world brings about a very strong drop in believability and comfort and may be rejected by human participants as a defense mechanism. In our present work, we have not incorporated a measure for quantifying the extent of realism. The design elements integrated into the VR task positively influenced or hindered the participant’s overall performance. In future, we intend to evaluate how the extent of realism and the integration of various design elements in a VR task affect the dynamic decision-making performance. In addition, we also intend to evaluate the neural dynamics [through neurophysiological measures like Electroencephalography (EEG) and functional near-infrared spectroscopy (fNIRS)] underlying VR training. This will enable us to understand and interpret how VR engages the proprioceptive senses and influences decision-making in the brain.

We found the percentage number of enemies killed significantly higher in both heterogenous and difficult conditions on Day 8 compared with the sham condition. These results were consistent with the instance-based learning theory (IBLT) as proposed by Gonzalez and Dutt (2011), and the retrieval effort hypothesis proposed by Pyc and Rawson (2009). As per IBLT (Gonzalez and Dutt, 2011), the participants in the heterogenous condition were able to store multiple instances of information regarding the behavior of the enemies in the environment during repetitive training. These numerous variable instances helped the participants quickly retrieve, process, and make quick and efficient decisions on the corresponding association between maneuvering through the environment and simultaneously shooting the enemies in quick time (Gonzalez and Dutt, 2011). As per the retrieval effort hypothesis (Pyc and Rawson, 2009), during training from Days 2 to 7, the participants in the difficult condition were able to make several difficult but successful retrievals of “if-then” procedures required to successfully negotiate through a search-and-shoot environment (Pyc and Rawson, 2009). Paired with immersive VR’s natural tendency to provide a sense of “presence,” participants could engage their proprioceptive senses to respond to the demands in the complex environment better (ter Haar, 2005). In addition, mixed factorial ANOVA revealed that participants in the heterogenous condition had a slight dip in the percentage number of enemies killed on Day 2, and participants in the difficult condition had a slight dip in the percentage number of enemies killed on Day 3. After these days, the percentage number of enemies killed in both the conditions kept on increasing till Day 8. This result could be explained through the skill, rules, and knowledge (SRK) taxonomy proposed by Rasmussen (1983). According to the SRK taxonomy, subjects in novel complex environments initially adopt knowledge-based behavior, which through repetitive training could eventually acquire rule-based behavior and subsequently skill-based behavior. According to Rasmussen (1983), the acquisition of knowledge-based behavior is very effortful and slow. During repetitive training in both the heterogeneous and difficult conditions, the participants slowly gathered information that led to the creation of rule-based heuristics in their brains, which eventually transformed into intuitive skill-based behavior by the end of the training period. Henceforth, on Day 8, the participants were able to adopt the SRK taxonomy in the baseline search-and-shoot environment, leading to enhanced performance as shown in the results. A similar pattern of results was observed for the accuracy index as well.

The results for the total time taken were quite interesting. The results from the mixed factorial ANOVA revealed that participants in both the heterogenous and the difficult conditions took significantly less time to complete/be terminated from the environment than the sham condition on Day 1. However, on Day 8, the participants in both the heterogenous and the difficult conditions recorded a significantly higher total time than the sham condition. One of the reasons could be that on Day 8, participants in both the heterogenous and difficult conditions were able to kill a greater number of enemies, which meant that they were able to traverse through different army bases in the environment and were able to survive in the environment much longer compared with the sham condition. Similar to the percentage number of enemies killed and the accuracy index and the total time taken also did not reveal any significant difference between the participants in the heterogenous and difficult conditions. The health index (which was indicative of the amount of health reduced per second) did not reveal any significant difference between participants in all three conditions.

The participants in the heterogenous and difficult conditions reported significantly lower mental demand on Day 8 compared with the sham condition. This result was consistent with IBLT proposed by Gonzalez and Dutt (2011), which argued that repetitive storage of successful, variable instances of ‘if-then’ procedures in the memory could significantly reduce the information processing requirements of a participant at transfer. This result was also consistent with Rasmussen’s (1983) SRK taxonomy, which implied that skill-based behavior was comprised of expected actions and directly connected with the search-and-shoot environment. Henceforth, participants in the heterogenous and difficult conditions were able to employ skill-based behavior on Day 8, due to the repetitive training obtained from Days 2 to 7, which eventually reduced their propensity to adopt knowledge-based behavior, which inherently had higher information processing requirements.

Other self-reported cognitive variables (like temporal demand, physical demand, and frustration level) did not reveal any significant difference between the different conditions. One reason for this insignificance might be due to the inherent nature of the task; there was no extraordinary physical exertion required to complete the objectives in the search-and-shoot environment. In addition, the time taken to execute the entire experimental protocol was quite limited, which eventually led to the participants not feeling frustrated.

Participants in the heterogenous and the difficult conditions also reported higher performance satisfaction than the sham condition on Day 8. This result implied that the participants in the heterogenous and difficult conditions were quite satisfied with the way they performed on Day 8 on the back of the respective repetitive training they undertook from Days 2 to 7. These results were consistent with the significantly higher performance measures recorded for both the heterogenous and difficult conditions on Day 8 than the sham condition. In addition, participants in both the heterogenous and difficult conditions reported significantly lesser effort required to achieve the level of performance seen on Day 8 compared with the sham condition. These results were also consistent with Gonzalez and Dutt’s (2011) IBLT and the successful acquisition of skill-based behavior through repetitive training as proposed by Rasmussen (1983). Interestingly, participants in the heterogenous condition reported significantly lesser effort required to execute the objectives in the search-and-shoot environment compared with the difficult condition on Day 8. One reason might be due to the acquisition of variable instances and procedures experienced during the training from Days 2 to 7 in the heterogenous condition. Since the baseline version of the environment executed by the participants on Day 1 and Day 8 was an amalgamation of the novice and difficult versions, participants in the heterogenous condition were able to efficiently allocate the memory for different instances encountered in both the conditions, subsequently leading to lesser effort required to retrieve those instances to make complex decisions on Day 8.

This article addresses the impact of different repetitive training frameworks on dynamic decision-making. As discussed before, most of the research in this domain has been directed toward generic motor tasks, language acquisition tasks, or simple memory tasks. All these tasks require the utilization of only basic cognitive processes. On the other hand, dynamic decision-making requires the effective encapsulation of different cognitive and sub-cognitive processes. One of the primary contributions of this article has been the successful evaluation of the efficacy of both heterogenous and difficult conditions in training personnel in novel and complex decision-making environments. These training frameworks can be adapted to train military personnel, industrial personnel, and medical personnel in immersive VR in complex environments. Even though this article has comprehensively evaluated the efficacy of two different repetitive training frameworks, other training frameworks like procedural reinstatement and cognitive antidote could also be evaluated for their efficiency in the transfer of cognitive skills in future. In addition, short-term and long-term transfer can also be assessed concerning all the aforementioned repetitive training frameworks to measure their propensity for long-term acquired skill retention. In addition, advanced feedback mechanisms, like brain-computer interfaces, haptic/olfactory feedback, and advanced AI could be incorporated into the immersive VR environments for providing more gratifying and realistic experiences significantly for training personnel.

## Data availability statement

The raw data supporting the conclusions of this article will be made available by the authors, without undue reservation.

## Ethics statement

The studies involving human participants were reviewed and approved by Institute Ethical Committee, Indian Institute of Technology Mandi. The patients/participants provided their written informed consent to participate in this study.

## Author contributions

AR contributed toward designing the simulation, the experiment and carried out the data collection, and analyses of the work. SC aided in the data collection for the experiment. VD was the principal investigator who developed the idea of the experiment and served as a constant guiding light for this work. All authors contributed to the article and approved the submitted version.
